# Small-scale spatial analysis of intermediate and definitive hosts of *Angiostrongylus cantonensis*

**DOI:** 10.1186/s40249-018-0482-8

**Published:** 2018-10-15

**Authors:** Qiu-An Hu, Yi Zhang, Yun-Hai Guo, Shan Lv, Shang Xia, He-Xiang Liu, Yuan Fang, Qin Liu, Dan Zhu, Qi-Ming Zhang, Chun-Li Yang, Guang-Yi Lin

**Affiliations:** 1 National Institute of Parasitic Diseases, Chinese Center for Disease Control and Prevention; Chinese Center for Tropical Diseases Research, Shanghai, 200025 China; 2 WHO Collaborating Centre for Tropical Diseases; National Center for International Research on Tropical Diseases, Ministry of Science and Technology, Shanghai, 200025 China; 3 Key Laboratory of Parasite and Vector Biology, Ministry of Health, Shanghai, 200025 China; 4 Centre for Disease Control and Prevention of Guangdong Province, Guangzhou, 510300 China; 5 Shanghai Medical College, Fudan University, Shanghai, 200032 China

**Keywords:** *Angiostrongylus cantonensis*, Snail intermediate host, *Pomacea canaliculata*, Rat definitive host, Spatial analysis

## Abstract

**Background:**

Angiostrongyliasis is a food-borne parasitic zoonosis. Human infection is caused by infection with the third-stage larvae of *Angiostrongylus cantonensis*. The life cycle of *A. cantonensis* involves rodents as definitive hosts and molluscs as intermediate hosts. This study aims to investigate on the infection status and characteristics of spatial distribution of these hosts, which are key components in the strategy for the prevention and control of angiostrongyliasis.

**Methods:**

Three villages from Nanao Island, Guangdong Province, China, were chosen as study area by stratified random sampling. The density and natural infection of *Pomacea canaliculata* and various rat species were surveyed every three months from December 2015 to September 2016, with spatial correlations of the positive *P. canaliculata* and the infection rates analysed by ArcGIS, scan statistics, ordinary least squares (OLS) and geographically weighted regression (GWR) models.

**Results:**

A total of 2192 *P. canaliculata* specimens were collected from the field, of which 1190 were randomly chosen to be examined for third-stage larvae of *A. cantonensis*. Seventy-two *Angiostrongylus*-infected snails were found, which represents a larval infection rate of 6.1% (72/1190). In total, 110 rats including 85 *Rattus norvegicus*, 10 *R. flavipectus*, one *R. losea* and 14 *Suncus murinus* were captured, and 32 individuals were positive (for adult worms), representing an infection rate of 29.1% of the definitive hosts (32/110). Worms were only found in *R. norvegicus* and *R. flavipectus,* representing a prevalence of 36.5% (31/85) and 10% (1/10), respectively in these species, but none in *R. losea* and *S. murinus*, despite testing as many as 32 of the latter species. Statistically, spatial correlation and spatial clusters in the spatial distribution of positive *P. canaliculata* and positive rats existed. Most of the spatial variability of the host infection rates came from spatial autocorrelation. Nine spatial clusters with respect to positive *P. canaliculata* were identified, but only two correlated to infection rates. The results show that corrected Akaike information criterion, *R*^2^, *R*^2^ adjusted and *σ*^2^ in the GWR model were superior to those in the OLS model.

**Conclusions:**

*P. canaliculata* and rats were widely distributed in Nanao Island and positive infection has also been found in the hosts, demonstrating that there was a risk of angiostrongyliasis in this region of China. The distribution of positive *P. canaliculata* and rats exhibited spatial correlation, and the GWR model had advantage over the OLS model in the spatial analysis of hosts of *A. cantonensis*.

**Electronic supplementary material:**

The online version of this article (10.1186/s40249-018-0482-8) contains supplementary material, which is available to authorized users.

## Multilingual absracts

Please see Additional file 1 for translations of the abstract into the five official working languages of the United Nations.

## Background

Angiostrongyliasis is a food-borne parasitic zoonosis while human infection is caused by infection with the third-stage larvae of *Angiostrongylus cantonensis*. The life cycle of *A. cantonensis* involves rodents as definitive hosts and molluscs as intermediate hosts [1]. Humans are dead-end hosts for *A. cantonensis*, and they can still be infected through ingestion of infected molluscs, contaminated vegetables, or transport hosts, such as shrimps, crabs, frogs and lizards [2]. Infected individuals present with eosinophilic meningitis or meningoencephalitis [3]. More than 30 recent outbreaks have been reported in mainland China so far [4–7]. Human infection is listed as an emerging food-borne parasitic disease by the World Health Organization (WHO) and is classified as an emerging infectious disease by the Chinese Ministry of Health since 2003 [6, 8, 9].

Previously, studies on angiostrongyliasis were mainly concentrated on epidemic foci and case descriptions. Most reports were based on descriptive epidemiological methods in general, while the spatial parameters were seldom considered [10]. In recent years, spatial analysis using the geographic information system (GIS) has started to be applied more widely in the field of parasitic diseases [11, 12]. From then on, recent such work on schistosomiasis and malaria at different scales has provided new ideas and methods for angiostrongyliasis research. It would be therefore important to know the spatial distribution, infection status, and the factors that influence these parameters both for snail intermediate hosts and for rat definitive hosts. Such knowledge would be most useful for the prevention and control of angiostrongyliasis and estimating risk of infection. This study aimed at analysing the characteristics of spatial distribution of these two hosts at village scale, by exploring the applicability of ordinary least squares regression (OLS) and geographically weighted regression (GWR) models to provide a methodology reference and theoretical basis for the prevention and control of this emerging human parasitic disease.

## Methods

### Study area

**Fig. 1:**
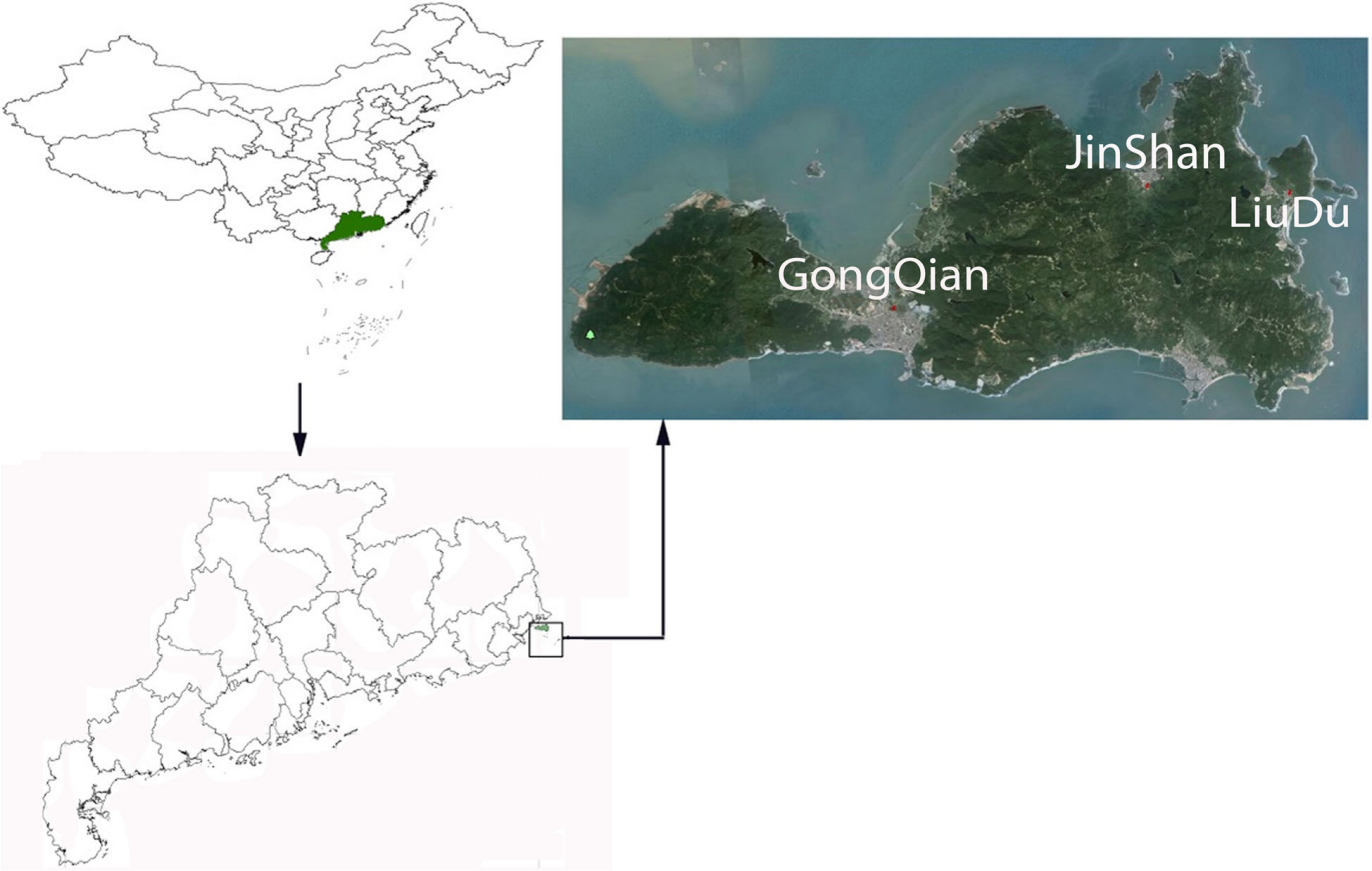
Geographical location of Nanao Island and the villages involved in the study

Nanao Island is located at the extreme eastern edge (116°53′–117°19′E, 23°11′–23°32′N) of Guangdong Province, China (see Fig. 1). The annual average temperature is 21.5 °C and the annual average rainfall is 1348 mm. The snail intermediate host *Pomacea canaliculata* and various rat definitive hosts of *A. cantonensis* are widely distributed on the island. Besides, from previous research data, there have been revelations that there might be hotspots of *A. cantonensis* infections in this region of China [13, 14].

### Data collection

Three villages on Nanao Island (Gongqian Village, Liudu Village and Jinshan Village) belonging to the towns of Houzhai, Shenao and Yunao, respectively, were selected as study areas for stratified random sampling. The density and infection status of *P. canaliculata* and various rat species were surveyed every three months from December 2015 to September 2016. The method of trapping rats was referred to *sampling procedure of vector infected by pathogens – Rodent* (GB/T28940–2012 China). In addition, environmental data, such as season, environmental type, distance from residential areas and fresh-water relation were also collected. The geographical coordinates for each sampling point were recorded by a Garmin 60CS (Garmin Corp., Olathe, KS, USA) global positioning system (GPS) instrument.

*Pomacea canaliculata* lung and muscles were examined for third-stage larvae by lung-microscopy, tissue homogenate and enzyme digestion [15]. Rats were captured by night feeding and trapping, and were drowned and dissected with hearts and lungs checked for adult worms. Morphological observation was performed for *P. canaliculata*, rats and third-stage larvae of *A. cantonensis*, followed by DNA extraction and polymerase chain reaction to double-check the species. A unique identification number for every sampling site, including spatial data, density and natural infection rate, was established to reflect the distribution and infection status of these hosts.

### Statistical analysis

#### Descriptive analysis

Infection rates and densities of snails and rats were analysed by non-parametric rank and summing tests using the statistical software SAS 9.3 (SAS Institute, Cary NC, USA) and the Chi-square test (Fisher’s exact test). The correlation between density and infection rate was analysed by Spearman’s rank correlation. The level of *P* < 0.05 (with two-tailed test) was chosen for statistical significance.

#### Spatial correlation analysis

ArcGIS (version 10.2, ESRI Corp., Redlands, CA, USA) was used to analyse the spatial correlation of the infection rates, both for snails and for rats, by establishing a semivariogram model, which provides the measure of variance as a function of distance between data points. The semivariance graph conveyed information about the continuity and spatial variability of the process [16, 17]:

$$ \mathrm{r}(h)=\frac{1}{2N(h)}{\sum}_{i=1}^{N(h)}{\left[Z\left({x}_i\right)-Z\left({x}_i+h\right)\right]}^2. $$

In the formula, Z(*x*_*i*_) and *Z*(*x*_*i*_ + *h*) are sample values at locations *x*_*i*_ and ( *x*_*i*_ + *h*), *N*(*h*) the number of paired data at the distance *h* (points with *h* separation distance), and r(h) the semivariance. The value of the semivariogram model is based on the fixed distance *h*. The model includes the following four parameters: Nugget, Range, Sill and Partial Sill. The degree of spatial autocorrelation is reflected by the ratio between Partial Sill and Sill; the greater the ratio, the stronger the spatial autocorrelation. The ratio between Nugget and Sill is called the substrate effect, which represents the variation among samples and is caused by a random factor.

#### Scan statistics

SaTScan 9.4 (https://www.satscan.org/) was used to detect spatial aggregation of infection rates. The parameters were set as Poisson distribution model and 30% of the population at risk as maximum spatial cluster size. Monte Carlo simulation was used to find the likelihood ratio (LLR) and to explore the maximum possible cluster [18–20].

#### Spatial modelling

The OLS and GWR models were compared with respect to their ability to analyse the relationship between season, type of environment, distance from residential areas and density on the one hand, and the spatial distribution of infection rates (both in the snail and in the rat definitive hosts) on the other hand. This was performed by using the *Geostatistical Analyst* tool in ArcGIS by comparing the degree of fit.

The OLS model is a global spatial analytical method where the basic assumption is that the dependent and independent variables have the same linear relations in all spatial parts of the area studied, i.e. its parameters are constant in different locations. Therefore, OLS can only produce average and global parameter estimates, rather than local ones [21]. The OLS model was calculated as follows:

$$ {y}_i={\beta}_0+{\sum}_{j=1}^n{\beta}_j{\chi}_{ij}+{\varepsilon}_i; $$

Where *y*_*i*_ is the value at the *i*^th^ region, *I* = 1, 2…N, *χ*_*ij*_ the *j*^th^ variable value at the *i*^th^ region, and *ε*_*i*_ the linear random error.

The GWR model is a local spatial analytical method, which is mainly used in non-stationary parameter estimation for the analysis of local spatial relation. Its parameters can change with different geographic positions. Since both spatial autocorrelation and spatial heterogeneity are taken into account in the GWR model, it has more value in the choice of spatial influence factors of disease compared with the general spatial models, such as OLS, spatial lag model, spatial error model and spatial Durbin model [22–24]. The GWR model was calculated as follows:

$$ {Y}_i={\beta}_0\left({u}_i,{\nu}_i\right)+{\sum}_{j=1}^k{\beta}_j\left({u}_i,{\nu}_i\right){\chi}_{ij}+{\varepsilon}_i; $$

where the term (*u*_*i*_, *ν*_*i*_)is the geographic coordinates of the *i*^th^ sample, *β*_*j*_ the regression coefficient that changes with geographic position, and *ε*_*i*_ the linear random error.

## Results

### Descriptive analysis

**Fig. 2:**
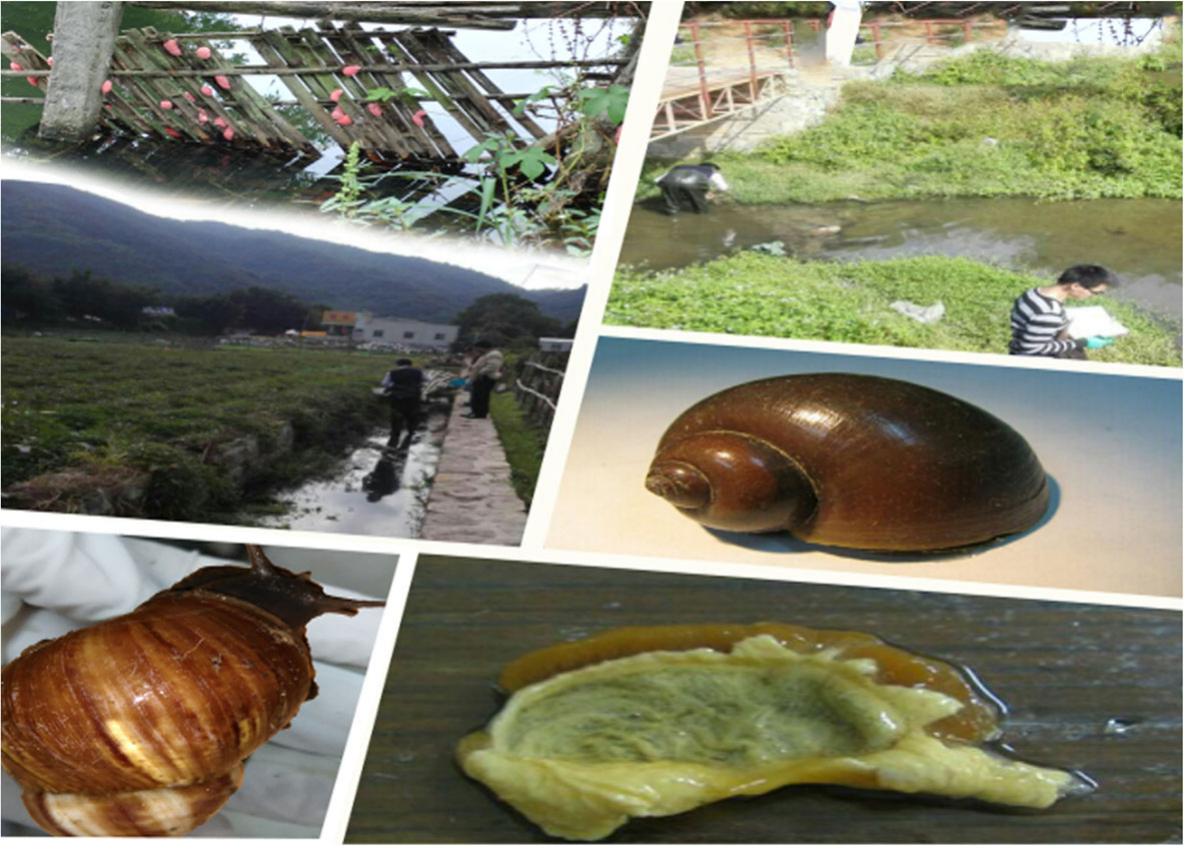
Collection of snails and screening of *Angiostrongylus cantonensis*

A total of 2192 *P. canaliculata* snails were collected from the three study villages (see Fig. 2). About half of the sails (1190) were randomly chosen to be microscopically examined, which resulted in 72 (6.1%) positives (see Table 1). When comparing according to the study sites, it was found that the difference in infection rates between the villages was statistically significant (*χ*^2^ **=** 12.8058, *P =* 0.0017). The infection rates between Gongqian Village and Jinshan Village were statistically significant (*χ*^2^ **=** 9.8581, *P =* 0.0017), as well as between Liudu Village and Jinshan Village (*χ*^2^ **=** 6.5297, *P =* 0.0106). However, the infection rates were not statistically significant (*χ*^2^ **=** 1.3459, *P =* 0.2460) between Gongqian Village and Liudu Village.

**Table 1:** Summary of the investigations on the snail intermediate hosts

Sampling village	Number of collected snails	Number of examined snails	Number of infected snails	Density (number/m^2^)	Infection rate (%)
Gongqian	644	283	9	8.00	3.18
Liudu	886	486	24	9.92	4.94
Jinshan	662	421	39	3.54	9.26
Total	2192	1190	72	6.66	6.05

**Fig. 3:**
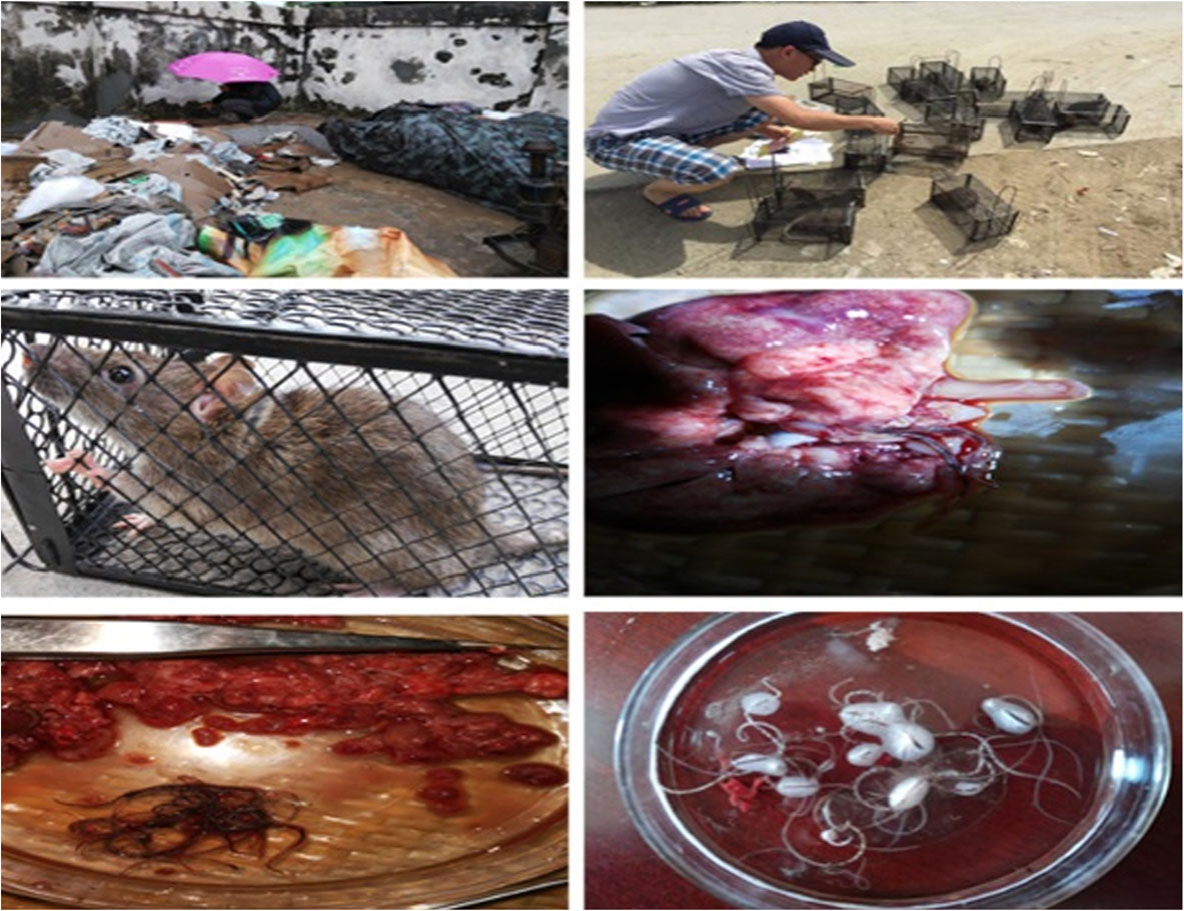
Capture of rats and screening of *Angiostrongylus cantonensis*

A total of 110 rats were captured, including *R. norvegicus*, *R. flavipectus* (see Fig. 3), *Rattus losea* and *Suncus murinus*, of which 32 (29.1%) were positive (see Table 2). There were 31 positive *R. norvegicus* and only one *R. flavipectus,* but no adult worms at all were found in *R. losea* and *S. murinus*. The variation of infection rate between different species of rodents was significant (Fisher’s Exact Test, *P =* 0.0051), and there was also a significant variation of the infection rate of rodents between the villages (*χ*^*2*^ **=** 13.9719, *P =* 0.0009). The infection rates between Gongqian Village and Jinshan Village were statistically significant (*χ*^*2*^ **=** 12.1951, *P =* 0.0005). However, the infection rates between Gongqian Village and Liudu Village (*χ*^*2*^ **=** 3.0590, *P =* 0.0803), as well as between Liudu Village and Jinshan Village (*χ*^*2*^ **=** 1.6250, *P =* 0.2024) were not statistically significant.

**Table 2:** Summary of the investigations on the rat definitive hosts

Sampling village	Number of mouse cages	Number of captured rats	Number of infected rats	Density (number/cage)	Infection rate (%)
Gongqian	289	71	29	0.25	40.85
Liudu	117	13	2	0.11	15.38
Jinshan	130	26	1	0.20	3.85
Total	536	110	32	0.21	29.09

The Spearman’s rank correlation tests showed that no statistically significant correlation was observed between *P. canaliculata* densities and infection rates (r_s_ = 0.20582, *P =* 0.2151), but the correlation between these parameters in the rats was significant (r_s_ = 0.51755, *P* ≤ 0.0001).

### Spatial autocorrelation

**Fig. 4:**
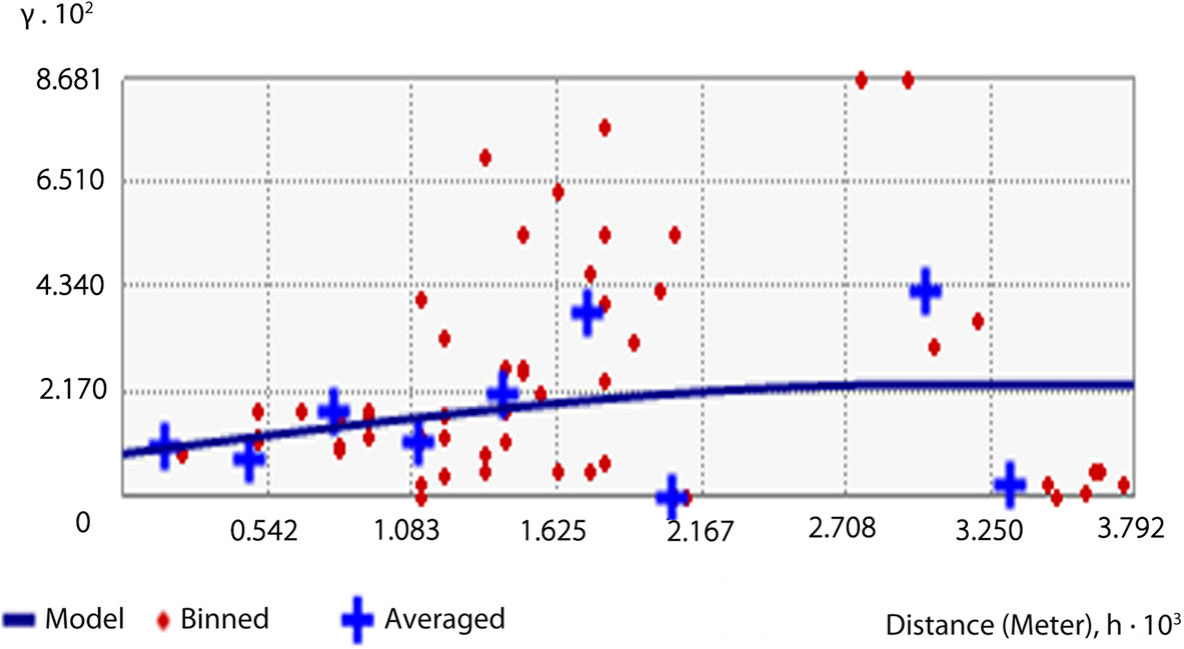
Semivariogram of infection rate of *Angiostrongylus cantonensis* in *Pomacea canaliculata*

The semivariance model for infection rate of *P. canaliculata* (see Fig. 4), was:

$$ \mathrm{r}(h)={0.008833}^{\ast}\mathrm{Nugget}+{0.014418}^{\ast}\mathrm{Spherical}(0.0030444). $$

In this model, the values of Nugget, Partial Sill and Sill were 0.008833, 0.014418 and 0.023251, respectively. The ratio of Nugget to Sill was 38% and the ratio of partial Sill to Sill was 62%, which indicated that the spatial heterogeneity was mainly caused by spatial autocorrelation.

**Fig. 5:**
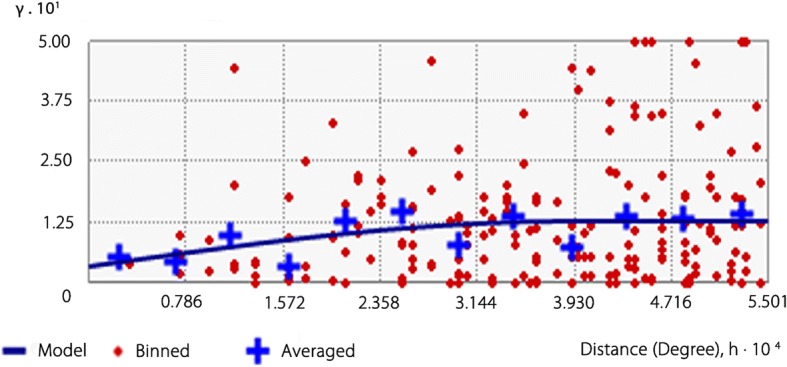
Semivariogram of infection rate of *Angiostrongylus cantonensis* in rats

The semivariance model for the infection rate of rats (see Fig. 5), was:

$$ \mathrm{r}(h)={0.032829}^{\ast}\mathrm{Nugget}+{0.093567}^{\ast}\mathrm{Spherical}(0.00038847). $$

Here, the value of Nugget, Partial Sill and Sill were 0.032829, 0.093567 and 0.093567, respectively. The ratio of Nugget to Sill was 26% and the ratio of partial Sill to Sill was 74%, which also indicated that the spatial heterogeneity was mainly caused by spatial autocorrelation.

### Spatial scan statistics

As shown in Tables 3 and 4, eleven spatial clusters were detected: nine clusters of infected snails and two of infected rats. The maximum radius of accumulation areas in the infection rates of *P. canaliculata* and rats were 0.049 km and 0.069 km, respectively.

**Table 3:** Scanning of *Angiostrongylus cantonensis* infection rate in *Pomacea canaliculata*

Cluster ID	Cluster centre	Radius (m)	Population (number)	Positives (number)	RR	LLR	*P*-value
1	23.454220 N, 117.094280 E	49	59	18	6.39	16.33	0.000
2	23.447210 N, 117.127850 E	2.4	53	12	4.29	7.63	0.003
3	23.454330 N, 117.100740 E	–	12	5	7.33	5.51	0.033
4	23.429340 N, 117.027880 E	–	29	6	3.64	3.26	0.300
5	23.453960 N, 117.092300 E	–	8	3	6.42	3.00	0.368
6	23.455660 N, 117.099530 E	–	23	4	2.98	1.66	0.893
7	23.455690 N, 117.101070 E	–	26	4	2.63	1.35	0.954
8	23.448060 N, 117.127360 E	7.1	61	7	1.99	1.25	0.970
9	23.448060 N, 117.127360 E	–	33	4	2.06	0.81	0.998

*RR* Relative risk, *LLR* Log likelihood ratio

**Table 4:** Scanning of *Angiostrongylus cantonensis* infection rate in rats

Cluster ID	Cluster centre	Radius (m)	Population (number)	Positives (number)	RR	LLR	*P*-value
1	23.429990 N, 117.026060 E	36	23	13	2.59	3.19	0.279
2	23.430870 N, 117.025420 E	69	27	12	1.84	1.32	0.955

*RR* Relative risk, *LLR* Log likelihood ratio

**Fig. 6:**
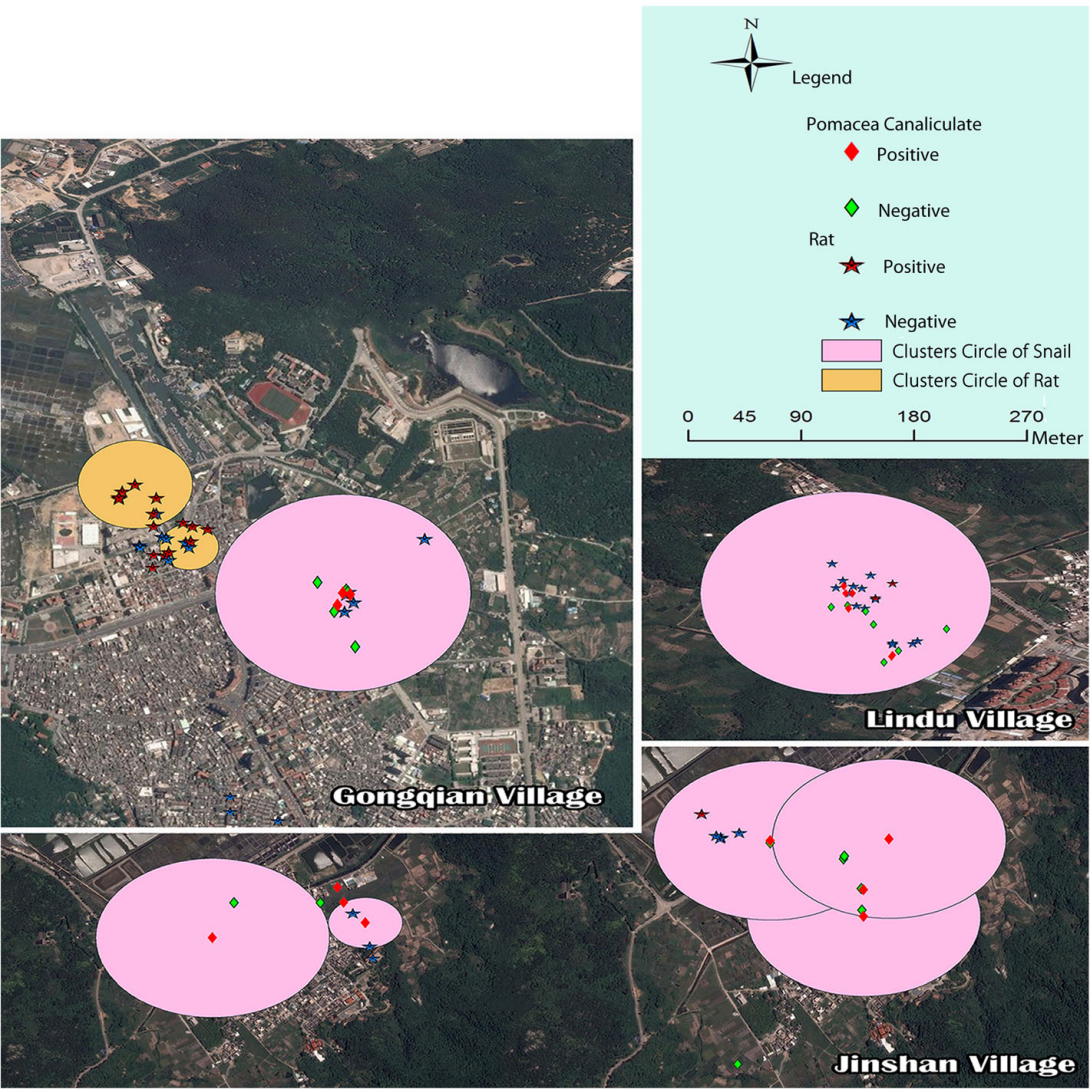
An overview of the sampling site and cluster areas of positive samples

Spatial cluster areas and the sampling sites were added to Remote Sensing maps from Google Earth for spatial overlay analysis. The infected snail clusters were mostly seen in artificial channels near the villages, while the infected rat clusters were found in places characterized by the presence of messy environment, such as rubbish heaps and recycling centres (see Fig. 6).

### Spatial modelling

First, the OLS model was selected to find the associations with the variables chosen and then the modelling was performed to decide whether the GWR model was also needed. The parameters and the fitting criterion of OLS model were obtained (see Table 5 and Table 6).

**Table 5:** Infection rates of *Pomacea canaliculata* with *Angiostrongylus cantonensis* using different variables estimated by OLS approach

Variable	Coefficient	SE	*T*	*P*	Robust_SE	Robust_*T*	Robust_*P*	VIF
Intercept	6.8602	8.1193	0.8449	0.4042	6.0446	1.1349	0.2646	–
Season	0.7449	0.5753	1.2948	0.2044	0.5014	1.4856	0.1469	1.0257
Environment	−0.2110	1.9554	− 0.1079	0.9148	1.7820	− 0.1184	0.9067	1.0460
Distance	−2.7890	2.2998	−1.2127	0.2339	1.9624	−1.4213	0.1646	1.0222
Density	0.1857	0.3042	0.6104	0.5458	0.2584	0.7185	0.4775	1.0614

*AIC* = 306.0523, *AIC*_C_ = 308.7620, *R*^2^ = 0.0864, *R*^2^ adjusted = 0.0243, *σ*^2^ = 154.6894

*OLS* Ordinary least squares, *SE* Standard error, *VIF* Variance inflation factor

**Table 6:** Infection rates of rats with *Angiostrongylus cantonensis* using different variables estimated by OLS approach

Variable	Coefficient	SE	*T*	*P*	Robust_SE	Robust_*T*	Robust_*P*	VIF
Intercept	−16.6792	16.3587	−1.0196	0.3128	10.3744	−1.6078	0.1142	–
Season	1.7161	1.4141	1.2136	0.2306	1.3195	1.3006	0.1994	1.3183
Environment	6.3547	3.9842	1.5950	0.1170	3.6805	1.7266	0.0904	1.1972
Distance	−3.1430	5.8266	−0.5394	0.5920	3.1362	−1.0022	0.3210	1.1584
Density	61.0019	25.8755	2.3575	0.0224*	25.5840	2.3844	0.0209*	1.3275

*AIC* = 529.8539, *AIC*_C_ = 531.6039, *R*^2^ = 0.2976, *R*^2^ adjusted = 0.2414, *σ*^2^ = 790.7322

*OLS* Ordinary least squares, *SE* Standard error, *VIF* Variance inflation factor

*Statistical significance

With the aim to analyse the residual error in the OLS model of the *P. canaliculata* infection rate and that of the rats, Jarque-Bera statistic (JB) was instituted. JB for *P. canaliculata* infection rate was 13.013 (*P =* 0.0015), and that of the rats 21.627 (*P =* 0.00002). They were obviously both statistically significant, which indicates that the residual error did not fit the hypothesis of normal distribution, i.e. data on the infection rate was non-stationary in space and there was spatial heterogeneity. This means that spatial information was not fully extracted by the OLS model.

It is important to set the weighting when GWR model was established. In order to make sure of the best bandwidth, Gaussian function was chosen for this function based on the principle of minimum corrected Akaike information criterion (*AIC*_C_). The parameters and fitting criterion of GWR models are shown in Tables 7 and 8.

**Table 7:** Infection rates of *Pomacea canaliculata* with *Angiostrongylus cantonensis* using different variables estimated by GWR approach

Variable	Min	P25	M	P75	Max	SD
Intercept	−0.0060	0.0807	0.0808	0.0888	0.0929	0.0381
Seasons	0.0070	0.0071	0.0075	0.0077	0.0113	0.0017
Environmental types	−0.0074	−0.0074	−0.0065	− 0.0058	0.0037	0.0043
Distance	−0.0308	−0.3016	− 0.0293	−0.0292	− 0.0031	0.0110
Density	−0.0008	0.0021	0.0023	0.0027	0.0028	0.0014

*R*^2^ = 0.1307, *R*^2^ adjusted = 0.1345, *AIC*_*C*_ = −30.9822, RSS = 0.4857, *σ*^2^ = 0.0171

GWR: Geographically weighted regression; P25: The 25th percentile; M: Median; P75: The 75th percentile; SD: Standard deviation

**Table 8:** Infection rates of rats with *Angiostrongylus cantonensis* using different variables estimated by GWR approach

Variable	Min	P25	M	P75	Max	SD
Intercept	−0.2600	− 0.2580	−0.2575	0.0395	0.1290	0.1668
Seasons	0.0019	0.0020	0.0379	0.0382	0.0383	0.0179
Environmental types	−0.0145	0.0266	0.0683	0.0684	0.0688	0.0303
Distance	−0.0833	−0.0831	− 0.0822	−0.0309	− 0.0307	0.0246
Density	−0.0560	0.0410	0.6670	0.6677	0.6703	0.3323

*R*^2^ = 0.4411, *R*^2^ adjusted = 0.3195, *AIC*_C_ = 27.0182, RSS = 3.1458, *σ*^2^ = 0.0709

GWR: Geographically weighted regression; P25: The 25th percentile; M: Median; P75: The 75th percentile; SD: Standard deviation

The results from the comparison of the OLS and the GWR approaches are displayed in Table 9. *AIC*_*C*_ (*AIC*_C_ = (2 k-2 L)/*n* + 2 k (k + 1)/(*n* – k – 1)) is a criteria to evaluate the performance of statistical model; where n was the sample size, k repeated the concision and L repeated the accuracy of the model, meaning that if *AIC* or *AIC*_C_ was smaller, the model was better. The coefficient of determination (*R*^2^) and the degree-of-freedom adjusted coefficient of determination (*R*^2^ adjusted) assumed that the independent variable explains the variation in the dependent variable in the model. The model was better if the value of *R*^2^ and *R*^2^ adjusted was more approximate to zero. Mean square deviation (*σ*^2^) and residual sum of squares (RSS) were the deviation of variables. Concerning *P. canaliculata,* for OLS model, *AIC* was 306.0523, *AIC*_C_ 308.7620, *R*^2^ 0.0864, *R*^2^ adjusted 0.0243, and *σ*^2^ 154.6894; while for GWR model, *AIC*_C_ was − 30.9822, *R*^2^ 0.1307, *R*^2^ adjusted 0.1345, RSS 0.4857, and *σ*^2^ 0.0171. The results showed that *AIC*_C_, *R*^*2*^, *R*^*2*^ adjusted and *σ*^*2*^ in the GWR model were superior to the ones in the OLS model for *P. canaliculata*. With regard to rats, for OLS model, *AIC* was 529.8539, *AIC*_C_ 531.6039, *R*^2^ 0.2976, *R*^2^ adjusted 0.2414, and *σ*^2^ 790.7322; while for GWR model, *R*^2^ was 0.4411, *R*^2^ adjusted 0.3195, *AIC*_C_ 27.0182, RSS 3.1458, and *σ*^2^ 0.0709. These also showed *AIC*_C_, *R*^*2*^, *R*^*2*^ adjusted and *σ*^2^ in the GWR model were superior to those in the OLS model for rats. According to some authors [25], the GWR approach should be chosen, even though it was more complex, if the difference of *AIC* between the two models were greater than 3. In our study, for both *P. canaliculata* and rats, *AIC*_C_ in the GWR models was much larger than the value in the OLS models, indicating that the GWR model had more advantage in analysing spatial heterogeneity data. However, *R*^2^ was not big in any of the two models, implying that the account of spatial variance that the models could explain was small, and the existing influence factor did not properly represent the spatial variance of infection rate data.

**Table 9:** Comparison between OLS and GWR models

Host	Approach	*AIC*_*C*_	*R* ^2^	*R*^2^ adjusted	*σ* ^2^
*Pomacea canaliculata*	OLS	308.7620	0.0864	0.0243	154.6894
	GWR	−30.9822	0.1307	0.1345	0.0171
Rat species	OLS	531.6039	0.2976	0.2414	790.7322
	GWR	27.0182	0.4411	0.3195	0.0709

*OLS* Ordinary least squares, *GWR* Geographically weighted regression, *AIC*_C_ Corrected Akaike information criterion

## Discussion

In comparison with some other cities/provinces in China, e.g., Shenzhen [26] and Xiamen [27] and Hainan [28], the infection rates of *P. canaliculata* were lower than those from our findings, but they were higher in Dali [29] and Guangzhou [30]. However, our reported rate of infected rats was lower than those reported in Rio de Janeiro in Brazil [31], Canary Islands [32] and Nueva Ecija in the Philippines [33]; while it was higher than those found in Guangzhou [34] and Zhongshan [35] in China.

There was a significant difference in the infection rate between the different kinds of rats investigated, e.g., only one infected *R. flavipectus* was found, while there was no infection in either *R. losea* or *S. murinus*. The reason could be attributed to the biological variation between different rat species, i.e. *R. norvegicus* and *R. flavipectus* live close to human dwellings and they were also known to prey on snails, while *R. losea* and *S. murinus* were field mice mainly feeding on plants [36], thereby lowering the expectation of infection.

The spatial distribution of infection rate of both *P. canaliculata* and the rat species showed spatial autocorrelation and aggregation. More so, positive *P. canaliculata* were mostly clustered in artificial channels near the villages, while the positive rats were more clustered in the places where the environment was what can be described as messy; consisting of garbage. Such places were always near human settlements, thus strengthening the probability for rats and molluscs to infect each other.

The result showed that GWR model was better than OLS model in terms of applicability, but the degree of fit of this model were not impressive. The possible reason was that our choice of factors that might influence infection was incomplete and some other possibly important factors, such as the normalized difference vegetation index (NDVI), temperature, rainfall, soil pH and socioeconomic status of the humans in the study area [37–40] were not part of our modelling. Such variables, particularly factors with potentially higher explanatory power need to be explored and included into spatial models in the future, in order to perfect further spatial models. We would also like to investigate different spatial scales [41], as well as improve the degree of fit of the models used by increasing sample sizes and the number of villages, with the expectation that these parameters might affect the finding.

## Conclusions

The intermediate hosts and definitive hosts of *A. cantonensis* were widely distributed in Nanao Island and positive infection has also been found in the hosts. These findings indicate that there was a risk of angiostrongyliasis in this region of China, and intensive monitoring work on the hosts should be undertaken.

Our study also showed that there existed spatial correlation and spatial clusters in the spatial distribution of positive *P. canaliculata* and rats. The maximum radius of spatial cluster areas of positive rats was basically consistent with the rats’ sphere of activity. More so, GWR model had advantage over OLS model in the spatial analysis of hosts of *Angiostrongylus cantonensis*.

## Additional file

Additional file 1: Multilingual abstracts in the five official working languages of the United Nations. (PDF 261 kb)

## Abbreviations

*AIC*: Akaike information criterion
*AIC*_C_: Corrected Akaike information criterion
GIS: Geographic Information System
GPS: Global positioning system
GWR: Geographically weighted regression
JB: Jarque-Bera
LLR: Log likelihood ratio
OLS: Ordinary least squares
RR: Relative risk
WHO: World Health Organization
