# Supplementary material for: Small-scale spatial analysis of intermediate and definitive hosts of Angiostrongylus cantonensis
Source: Infect Dis Poverty. 2018 Oct 15;7:100. doi: 10.1186/s40249-018-0482-8 (PMC6192004; doi:10.1186/s40249-018-0482-8)
Supplement: Supplementary file 1 — Multilingual abstracts in the five official working languages of the United Nations. (PDF 261 kb) [file 40249_2018_482_MOESM1_ESM.pdf]

Translation of the abstract into the five official working languages of the United Nations

تحليل مكاني ضيق النطاق للمضيف الوسيط والنهائي لداء الأسطوانية الكنتونية

تشيو آن خو، إي جانغ، يين هاي قوا، شان آل في،شان شيا، خا-شيانغ ليو، يآن فان، تشين ليو، تان جو، تشي- مين جانغ، تشون- لي يان و قوان- يي لين◄

الملخص

الخلفية:داء الأسطوانيات من الأمراض الحيوانية الطفيلية المتناقلة عن طريق الأغذية، وتسبب عدوى للإنسان عن طريق العدوى باليرقات من الطور الثالث من الإسطوانية الكنتونية . تتضمن دورة حياة الطفيليات القوارض كمضيف نهائي والرخويات كمضيف متوسط. تهدف هذه الدراسة إلى التحقق من حالة الإصابة وخصائص التوزيع المكاني لهذه المضيفات، بإعتبارهم المكون الرئيسي في إستراتيجية الوقاية ومكافحة داء الأسطوانيات.

الطرق:تم اختيار ثلاث قرى من جزيرة ناناو، ومقاطعة قوان دو، الصين، كمناطق دراسة عن طريق أخذ عينات عشوائية طبقية. حيث تم معاينة معدل الكثافة والإصابة الطبيعية بحلزون التفاح الذهبي" Pomacea canaliculata" كل ثلاث أشهر من ديسمبر/ كانون الأول2015 إلى غاية سبتمبر/ أيلول 2016، ذات الروابط المكانية الإيجابيةلحلزون التفاح الذهبي ومعدل الإصابة من خلال استخدام نظم المعلومات الجغرافي و المسح الإحصائي والمربعات الصغرى الخطية (OLS) ونمودج الإنحدار الجغرافي (GWR).

النتيجة : تم جمع مجموع 2192عينة منحلزون التفاح الذهبيمن الحقول، أُختير منهم عشوائيا 1190 بغرض فحصه من إحتمال إصابتهم بيرقات من الطور الثالث من الإسطوانية الكنتونية . تم إيجاد 27 حلزونمصاب بداء الأسطوانيات، والذي يمثل معدل الإصابة باليرقانة بنسبة 6.1% (1,190/72). المجموع: تم الإمساك بـ110 فأر من بينهم 85 جرذ نرويجي، و10جرذان من جنس فلافي بيكتوسو واحد من جنس راتوس لوسياو14 جرذ آسيوي"Suncus murinus" وقد وُجد 32 حالة إيجابية (للديدان البالغة)، ويمثل معدل الإصابة بـ29.1% كمضيفات نهائية(110/32). تم إيجاد الديدان فقط في الجرذان النرويجية وجرذان فلافي بيكتوس"flavipectus" مما يمثل إنتشارا بنسبة 36.5% (85/31) و 10% (10/1)على التوالي في هذه الأصناف، ولم يوجد أي منها فيجرذان راتوس لوسيا وموريس، بالرغم من اختبار 32 حالة من الأصناف الأخيرة. ومن الناحية الإحصائية، تم إيجاد روابط مكانية وعناقيد مكانية في التوزيع المكاني إيجابية عند حلزون التفاح الذهبي وعند الفئران. معظم التباين المكاني لمعدلات عدوى المضيف جاءت من الترابط الذاتي المكاني. وتم تحديد تسعة تجمعات مكانية إيجابية فيما يتعلق بـحلزون التفاح الذهبي ولكن حالتين فقط مرتبطة بمعدلات الإصابة. كما أظهرت النتائج أن معيار $_C$AIC ت، وR^2، و^2R معتدلة و σ^2 في نموذج الإنحدار الجغرافي " GWR" كان أعلى من نمودج المربعات الصغرى الخطية"OLS".

النتيجة : تم إيجاد حلزون التفاح الذهبي والجرذان موزع بشكل واسع في جزيرة ناناو، و تم إيجاد نتيجة إيجابية لإصابة المضيف، مما يؤكد خطر الإسطوانيات في هذه المناطق من الصين . وأظهر التوزيع الإيجابي لحلزون التفاح الذهبي والجرذان ارتباطًا مكانيًا، وكان لنموذج الإنحدار الجغرافي" GWR"ميزة على نموذج المربعات الصغرى الخطية"OLS" في التحليل المكاني لمضيف الإسطوانية الكنتونية.

Translated from English version into Arabic by Hadjela Amina and Aya Kurdi, through

TRANSLATORS
WITHOUT BORDERS

广州管圆线虫中间及终末宿主小尺度空间分析

胡求安，张仪，郭云海，吕山，夏尚，刘和香，方圆，刘琴，朱丹，张启明，杨春利，林光一

摘要

引言：广州管圆线虫病是由广州管圆线虫三期幼虫感染而引起的一种食源性寄生虫病。广州管圆线虫的生活史中间宿主为软体动物类，终末宿主为啮齿类动物类。本研究旨在调查广东省南澳岛上广州管圆线虫的中间宿主和终末宿主的密度及其感染现状，从而为该地区广州管圆线虫病的防治提供依据和策略。
方法：采用分层随机抽样法从南澳岛抽取 3 个行政村作为研究区域，分别于 2015 年 12 月～2016 年 9 月每个季度对南澳岛广州管圆线虫的中间宿主小管福寿螺和终末宿主鼠类进行监测调查，调查小管福寿螺和鼠类的分布及其感染状况，并借助于 ArcGIS、空间扫描统计以及最小二乘法（Ordinary Least Squares, OLS）模型和地理加权回归（Geographically Weighted Regression, GWR）模型对小管福寿螺和鼠类感染率进行空间自相关性和空间异质性分析。
结果：共采获 2192 只小管福寿螺，随机选取 1190 只用于检测广州管圆线虫三期幼虫感染情况。共检测到 72 只阳性小管福寿螺，感染率为 6.1% (72/1190)。共捕获鼠类 110 只，包括褐家鼠（Rattus norvegicus）85 只、黄胸鼠（Rattus flavipectus）10 只 、黄毛鼠（Rattus losea）1 只和臭鼩（Suncus murinus）14 只。共检测到阳性鼠 32 只，阳性感染率为 29.1% (32/110)。虫体只发现于褐家鼠和黄胸鼠中，感染率分别为 36.5% (31/85) 和 10% (1/10)，黄毛鼠和臭鼩没有检测到虫体感染。感染性小管福寿螺和感染性鼠的空间分布存在空间自相关性和空间聚集性，且其空间变异性主要来自空间自相关性。小管福寿螺检测到 9 个空间聚集区，鼠类检测到 2 个空间聚集区。此外，纠正 Akaike 信息标准（AIC_C），决定系数（R^2），调整性决定系数(R^2 adjusted) 和 均方差(σ^2) 均表明 GWR 模型优于 OLS 模型。

Translated from English version into Chinese by Qiu’an Hu

Analyse spatiale à petite échelle des hôtes intermédiaires et définitifs du nématode *Angiostrongylus cantonensis*

Qiu’an Hu, Yi Zhang, Yun-Hai Guo, Shan Lv, Shang Xia, He-Xiang Liu, Yuan Fang, Qin Liu, Dan Zhu, Qi-Ming Zhang, Chun-Li Yang and Guang-Yi Lin

Résumé
Contexte : L'angiostrongylose est une zoonose parasitaire d'origine alimentaire et l'infection humaine résulte d'une infection par les larves de troisième stade (L3) du nématode *Angiostrongylus cantonensis*. Le cycle de vie du parasite implique les rongeurs comme hôtes définitifs et les mollusques comme hôtes intermédiaires. Cette étude vise à étudier le stade d'infection et les caractéristiques de la distribution spatiale de ces hôtes, éléments clés de la stratégie de prévention et de contrôle de l'angiostrongylose.
Méthodes : Trois villages de l'île de Nanao, situés dans la province du Guangdong en Chine, ont été choisis comme zone d'étude par échantillonnage aléatoire stratifié. La densité et l'infection naturelle de *Pomacea canaliculata* et diverses espèces de rats ont été examinées tous les trois mois à partir de décembre 2015 jusqu'à septembre 2016, avec des corrélations spatiales du *P. canaliculata* positif et ArcGIS (système d'information géographique), des statistiques d'analyse, la méthode des moindres carrés ordinaires (MCO) et les modèles de régression géographiquement pondérée (GWR) ont permis l'analyse des taux d'infection.
Résultats : Un total de 2 192 spécimens de *P. canaliculata* ont été prélevés sur le terrain, dont 1 190 ont été choisis au hasard pour l'examen des larves L3 de *A. cantonensis*. Soixante-douze

escargots infectés par *Angiostrongylus* ont été retrouvés, ce qui représente un taux d'infection larvaire de 6,1% (72/1 190). Au total, 110 rats dont 85 *Rattus norvegicus*, 10 *Rattus flavipectus*, un *Rattus losea* et 14 *Suncus murinus* ont été capturés. 32 étaient positifs (pour les vers adultes), représentant un taux d'infection de 29,1% des hôtes définitifs (32/110). Les vers n'ont été trouvés que chez *R. norvegicus* et chez *R. flavipectus*, représentant respectivement une prévalence de 36,5% (31/85) et 10% (1/10) chez ces espèces, mais aucune chez *R. losea* et chez *S. murinus*, malgré des tests sur pas moins de 32 individus de cette dernière espèce. Statistiquement parlant, il existait une corrélation spatiale et des grappes spatiales dans la distribution spatiale des *P. canaliculata* et des rats positifs. L'essentiel de la variabilité spatiale des taux d'infection de l'hôte provient de l'autocorrélation spatiale. En ce qui concerne le *P. canaliculata* positif, neuf grappes spatiales ont été identifiées, avec seulement deux liées aux taux d'infection. Les résultats indiquent que les valeurs AIC_C, R^2, R^2 ajusté et σ^2 dans le modèle GWR étaient supérieures à celles du modèle MCO.
Conclusions : La vaste distribution du nématode *P. canaliculata* et des rats sur l'île de Nanao ainsi qu'une infection positive observée chez les hôtes démontrent l'existence d'un risque d'angiostrongylose dans cette région de Chine. La distribution du *P. canaliculata* et des rats positifs indiquait une corrélation spatiale et le modèle GWR présentait un avantage par rapport au modèle MCO dans l'analyse spatiale des hôtes de *A. cantonensis*.

Translated from English version into French by Suzanne Assenat and Fane Wann, through

TRANSLATORS
WITHOUT BORDERS

Маломасштабный пространственный анализ промежуточных и окончательных хозяев паразита *Angiostrongylus cantonensis*

Цю'ань Ху, И Чжан, Юнь-Хай Го, Шань Лу, Шан Ся, Хэ-Сян Лю, Юань Фан, Цинь Лю, Дань Чжу, Ци-Мин Чжан, Чунь-Ли Ян и Гуан-И Линь

Аннотация

Краткое описание: Ангиостронгилез представляет собой пищевой паразитарный зооноз и инфицирование людей происходит путем заражения личинками третьей стадии паразита *Angiostrongylus cantonensis*. В жизненном цикле паразита грызуны выполняют функцию окончательных хозяев, а промежуточными хозяевами служат моллюски. Данное исследование было направлено на изучение состояния инфекции и характеристик пространственного распределения этих хозяев, которые являются ключевыми компонентами стратегии по предотвращению и борьбе с ангиостронгилезом.
Методы: Для проведения исследования методом стратифицированного случайного отбора проб были выбраны три деревни на острове Нанао в провинции Гуандун, Китай. Каждые три месяца с декабря 2015 года по сентябрь 2016 года проводилось обследование плотности и природной инфекции улиток *Pomacea canaliculata* и различных видов крыс с пространственной корреляцией с зараженными улитками *P. canaliculata*. Уровень

зараженности анализировался при помощи ArcGIS (географической информационной системы), статистики сканирования, обычного метода наименьших квадратов (OLS) и моделей географически взвешенной регрессии (GWR).

Результаты: В полевых условиях было собрано всего 2192 образца *P. canaliculata,* из которых случайным образом было выбрано 1190 образцов для анализа на личинки третьей стадии *A. cantonensis*. Были найдены семьдесят две инфицированные улитки *Angiostrongylus,* что составляет уровень зараженности личинками 6,1% (72/1190). Было отловлено всего 110 крыс, включая 85 *Rattus norvegicus,* 10 *Rattus flavipectus*, одну *Rattus losea* и 14 *Suncus murinus.* Из них 32 особи были инфицированы (взрослыми червями), что составляет уровень зараженности окончательных хозяев 29,1% (32/110). Черви были обнаружены только в видах *R. norvegicus* и *R. flavipectus,* которые явились превалирующими с показателями 36,5% (31/85) и 10% (1/10) соответственно; однако, крысы *R. losea* и *S. murinus* не были инфицированы несмотря на тестирование 32 особей этих видов. Статистически существует пространственная корреляция и пространственные кластеры в пространственном распределении инфицированных улиток *P. canaliculata* и инфицированных крыс. В большинстве случаев пространственная изменчивость уровней зараженности хозяев обуславливалась пространственной автокорреляцией. Было выявлено девять пространственных кластеров для инфицированных улиток *P. canaliculata,* но только два кластера коррелировались с уровнями зараженности. Результаты показывают, что AIC_C, R^2, скорректированный R^2 и σ^2 в модели GWR превосходили те же коэффициенты в модели OLS.

Выводы: *P. canaliculata* и крысы широко распространены на острове Нанао и среди этих хозяев были также найдены инфицированные особи, что указывает на риск заболевания ангиостронгилезом в этом регионе Китая. Для распределения инфицированных улиток *P. canaliculata* и крыс характерна пространственная корреляция и модель GWR обладает преимуществом по сравнению с моделью OLS при пространственном анализе хозяев *A. cantonensis*.

Translated from English version into Russian by Natalia Potashnik and Alexander Somin, through

TRANSLATORS
WITHOUT BORDERS

Análisis espacial a pequeña escala de los huéspedes intermediarios y finales del *Angiostrongylus cantonensis*

Autores: Qiu’an Hu, Yi Zhang, Yun-Hai Guo, Shan Lv, Shang Xia, He-Xiang Liu, Yuan Fang, Qin Liu, Dan Zhu, Qi-Ming Zhang, Chun-Li Yang y Guang-Yi Lin

Resumen

Contexto: La angiostrongiliasis es una zoonosis parasitaria transmitida por el consumo de alimentos. La fase infectiva para el ser humano es la tercera fase larvaria del *Angiostrongylus cantonensis*. El ciclo vital de este parásito se desarrolla en roedores como huéspedes finales y moluscos como

intermediarios. Este estudio tiene como finalidad investigar el estado de la infección y las características de la distribución espacial de dichos huéspedes, ambos componentes esenciales de la estrategia a seguir para la prevención y el control de la angiostrongiliasis.

Metodología: Escogimos tres pueblos de la isla de Nanao, en la provincia china de Guangdong, que estudiamos mediante muestras aletorias estratificadas. Desde diciembre del 2015 hasta septiembre del 2016, examinamos cada tres meses la densidad y la infección natural de *Pomacea canaliculata* y otras especies de rata, con correlaciones espaciales de la *Pomacea canaliculata* positiva y los índices de infección analizados por ArcGIS (un sistema de información geográfica), estadísticas derivadas de escáneres, modelos de regresión de mínimos cuadrados ordinarios (OLS) y ponderados geográficamente (GWR).

Resultados: Se recogieron del campo un total de 2192 especímenes de *Pomacea canaliculata*, de los cuales 1190 fueron seleccionados aleatoriamente para el estudio de las larvas en tercera fase de *Angiostrongylus cantonensis*. Se encontraron 72 caracoles infectados de *Angiostrongylus*, lo cual constituye un índice de infección larval del 6,1 % (72/1190). En total, de las 110 ratas que capturamos (85 *Rattus norvegicus*, 10 *Rattus flavipectus*, una *Rattus losea* y 14 *Suncus murinus*), 32 individuos presentaban larvas adultas; un índice de infección del 29,1 % en los huéspedes finales (32/110). Las larvas se encontraron solo en las *Rattus norvegicus* y las *Rattus flavipectus*, lo que representa una prevalencia de, respectivamente, un 36,5 % (31/85) y un 10 % (1/10) en estas especies y de ninguna en las *Rattus losea* y *Suncus murinus*, pese a haber analizado hasta 32 individuos de estas especies. Estadísticamente, existía una correlación y agrupación espacial en la distribución espacial de individuos de *Pomacea canaliculata* infectados y de ratas infectadas. Gran parte de la variabilidad espacial de los índices de infección de los huéspedes se derivaba de la correlación espacial. Se identificaron 9 grupos espaciales con respecto a las *Pomacea canaliculata* positivas, pero solo dos guardaban correlación con los índices de infección. Los resultados arrojan que la AIC_C, R^2, R^2 ajustada y σ^2 en el modelo de regresión GWR eran superiores a los del modelo OLS.

Conclusiones: Las*Pomacea canaliculata* y las ratas estaban distribuidas por toda la isla de Nanao y los huéspedes también dieron positivo en la infección, lo cual demuestra que existe riesgo de angiostrongiliasis en esta zona de China. La distribución de la *Pomacea canaliculata* y las ratas mostraba una correlación espacial, y el modelo GWR mostró ser más eficaz que el OLS para el análisis espacial de los huéspedes de *Angiostrongylus cantonensis*.

Translated from English version into Spanish by Marta Callava Linares and Facundo Pagano, through

TRANSLATORS
WITHOUT BORDERS
